# Paradoxical expression of cell cycle inhibitor p27 in endometrioid adenocarcinoma of the uterine corpus – correlation with proliferation and clinicopathological parameters

**DOI:** 10.1038/sj.bjc.6600434

**Published:** 2002-07-15

**Authors:** J Watanabe, H Sato, T Kanai, Y Kamata, T Jobo, H Hata, T Fujisawa, E Ohno, T Kameya, H Kuramoto

**Affiliations:** Department of Pathology, Kitasato University School of Medicine, 1-15-1, Kitasato, Sagamihara, Kanagawa, 228-8555, Japan; Department of Clinical Cytology, Kitasato University Graduate School of Medical Sciences, 1-15-1, Kitasato, Sagamihara, Kanagawa, 228-8555, Japan; Department of Obstetrics and Gynecology, Kitasato University School of Medicine, 1-15-1, Kitasato, Sagamihara, Kanagawa, 228-8555, Japan; Department of Clinical Cytology, Kitasato University School of Allied Health Sciences, 1-15-1, Kitasato, Sagamihara, Kanagawa, 228-8555, Japan

**Keywords:** p27, endometrium, endometrioid adenocarcinoma, cell proliferation, clinicopathological parameter

## Abstract

p27 is regarded as a cyclin-dependent kinase inhibitor of the G1-to-S cell cycle progression by suppressing the kinase activity of cyclin/cyclin-dependent kinase complex. This study aimed to investigate p27 expression in the normal endometrium and endometrioid adenocarcinoma of the uterine corpus and the correlation of its expression with cell proliferation and clinicopathological parameters. Tissue samples of 127 endometrioid adenocarcinomas and 15 normal endometria were used in the study. Immunohistochemical staining for detecting p27 and Ki-67 was performed by the labelled streptavidin-biotin method on formalin-fixed and paraffin-embedded tissue samples. The expression was given as the labelling index, which indicates the percentage of positive nuclei. p27 staining was observed in the nuclei of the glandular cells in the functional layer of the secretory phase endometrium, whereas it was negative in those of the proliferative phase. In endometrioid adenocarcinomas, the labelling index of p27 expression paradoxically increased more significantly in the higher histological grades and was correlated with that of Ki-67. The high level of p27 expression was associated with clinicopathological parameters such as FIGO stage, lymph node metastasis, lymphovascular space involvement and myometrial invasion. High p27 expression was linked to higher grades of endometrioid adenocarcinoma, cell proliferation and some clinical prognostic factors. These results indicate that p27 might be an indicator of poor prognosis.

*British Journal of Cancer* (2002) **87**, 81–85. doi:10.1038/sj.bjc.6600434 www.bjcancer.com

© 2002 Cancer Research UK

## 

Cyclin-dependent kinase inhibitor (CKI) inhibits the cell cycle progression by suppressing the kinase activity of a variety of cyclin/cyclin-dependent kinase (cdk) complexes (Sherr, 1996). Abnormal expression of CKI is thought to play an important role in tumorigenesis and the progress of tumours.

CKIs are classified into two major families; the Ink4 family and the Cip/Kip family. P27 belongs to the Cip/Kip family and binds preferentially to the cyclin E/cdk2 and cyclin D/cdk4 complexes, and inhibits the G1-to-S progression (Russo *et al*, 1996). p27 knock-out mice sustain a variety of abnormalities including increased body size, multiple organ hyperplasia, pituitary tumour, and sterility due to underdeveloped ovaries (Nakayama *et al*, 1996). Therefore, p27 is predicted to act as a tumour suppressor.

It has been generally accepted that decreased p27 expression is related to poor prognosis in tumours of different histogenesis including lung (Kawana *et al*, 1998), oesophageal (Singh *et al*, 1998), colon (Loda *et al*, 1997), breast (Gillett *et al*, 1999), ovarian (Masciullo *et al*, 1999) and prostate cancers (Yanping *et al*, 1997).In contrast, it has also been reported that increased p27 expression is correlated with poor prognosis of the oesophagus (Anayama *et al*, 1998) and colon carcinoma (Cheng *et al*, 1999). In fact, it is not clear whether p27 expression is correlated positively or inversely with prognosis.

In the endometrium, it was reported that p27 was expressed in the secretory phase, but less in the proliferative phase (Shiozawa *et al*, 1998; Bamberger *et al*, 1999). Its expression was also higher in endometrial hyperplasia than in the proliferative phase and was significantly increased when the former was treated with medroxyprogesterone acetate (Shiozawa *et al*, 1998). Two studies mentioned that p27 was strongly reduced in endometrial carcinoma (Ahn *et al*, 1998; Bamberger *et al*, 1999), whereas a recent study showed a trend of increased p27 protein staining with high grade endometrial carcinoma (Nycum *et al*, 2001). p27 expression in endometrial carcinoma still remains contradictory. This study aimed to investigate p27 expression by immunohistochemical staining and the correlation of its expression with histological grades, cell proliferation and clinicopathological parameters in endometrioid adenocarcinoma of the uterine corpus.

## MATERIALS AND METHODS

### Materials

Tissue samples of 127 cases of endometrioid adenocarcinomas consisting of 73 well-differentiated adenocarcinomas (G1), 26 moderately-differentiated ones (G2), 28 poorly-differentiated ones (G3)) and 15 cases of normal endometrium (five with proliferative phase, 10 with secretory phase) were surgically obtained at Kitasato University Hospital between 1986 and 2000. Informed consent was obtained from all of the patients. Pathological diagnosis was classified according to the FIGO classification (FIGO, 1989). The patients' ages ranged from 30 to 83 years with the median age of 56 years. No patients had received either adjuvant chemotherapy or radiotherapy before surgery.

### Immunohistochemistry

Immunohistochemical staining for p27 and Ki-67 were performed with the labelled streptavidin-biotin method (DAKO, Kyoto, Japan). Tissue samples fixed in 10% formalin and embedded in paraffin were sectioned to 3 μm and deparaffinized in xylene. Endogenous peroxidase activity was blocked by 3% hydrogen peroxide for 30 min. Then, antigen retrieval was performed by autoclave at 121°C for 15 min in 0.1 mM citrate buffer (pH 6.0). After being incubated with 10% normal swine serum for 30 min, mouse monoclonal anti-human p27 antibody (clone 1B4, 1 : 200, Novocastra, Newcastle, UK) and rabbit polyclonal anti-human Ki-67 antibody (1 : 50 DAKO, Kyoto, Japan) were applied to the slides overnight at 4°C, respectively. As negative controls, normal mouse or rabbit serum at the same dilution was used. Diaminobenzidine reaction was done for visualisation of the signal and Mayer's haematoxylin was used for counterstaining.

The percentage of positive nuclear staining of p27 was evaluated by counting at least 1200 cells in high power fields independently by two observers and expressed as the labelling index (LI). They were blinded as to the pathological parameters. Interobserver variation was addressed by averaging the individual values. Its variation usually did not differ by more than 10%.

### Measurement of oestrogen receptor (ER) and progesterone receptor (PR)

Oestrogen receptor (ER) and progesterone receptor (PR) in tumour tissue freshly obtained at surgery were measured using the radioreceptor assay system of Kitasato Biochemical Laboratory (Kanagawa, Japan). ER and PR were defined as positive when gross bound count/non-specific bound count >2, Kd<1 nM l^−1^ and (straight line recurrence coefficient) >0.9.

### Statistical analysis of immunostaining

Statistical analysis of the correlation between LI of p27 and Ki-67 in the same patient was conducted by the Spearmann rank correlation test. The Mann–Whitney *U*-test was used to examine the correlation between LI of p27 and clinicopathological parameters. *P*-values less than 0.05 were considered significant.

## RESULTS

### p27 expression in normal endometria

**Figure 1:**
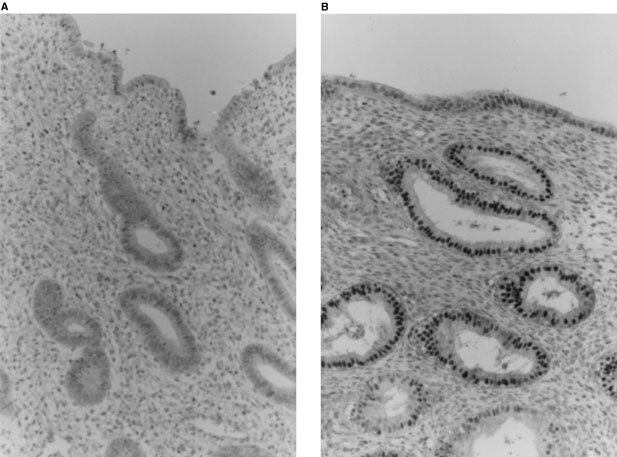
Immunohistochemical staining of p27 in normal endometrium. In the proliferative phase, the nuclei of the glandular cells in the functional layer are negative for p27 (**A**). In the secretory phase, the nuclei of the superficial lining cells and the glandular cells in the functional layer show positive for p27 (**B**). (Original magnification, ×200).

In the proliferative phase, the glandular cells in the functional layer were negligible for p27 (Figure 1A

). In the secretory phase, the nuclei of the superficial lining cells and the glandular cells of the functional layer were positive for p27 (Figure 1B).

### p27 expression in endometrioid adenocarcinomas

p27 staining was positive in the nuclei of endometrioid adenocarcinomas and the positive rate of all cases was 97.6% (124 out of 127 cases). The LIs of p27 expression in G1, G2 and G3 of endometrioid adenocarcinomas were 55.1±25.0, 64.4±15.1 and 75.0±10.7%, respectively. The LIs increased significantly in the higher histological grades (Table 1

**Table 1:**
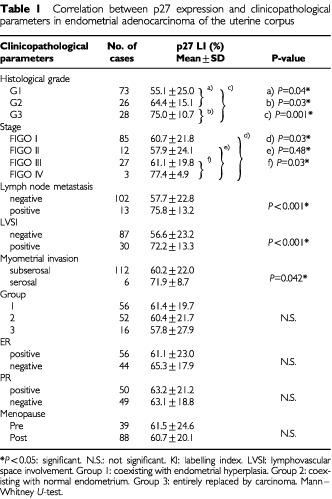
Correlation between p27 expression and clinicopathological parameters in endometrial adenocarcinoma of the uterine corpus

); Figure 2A,B

**Figure 2:**
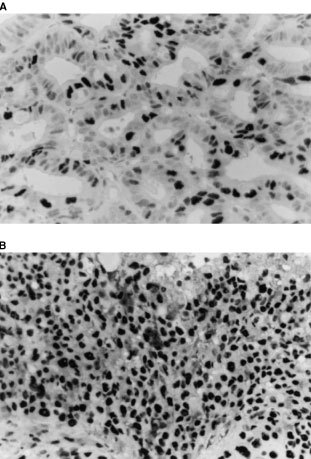
Immunohistochemical staining of p27 in endometrioid adenocarcinoma. The nuclei of G1 (**A**) and G3 (**B**) of endometrioid adenocarcinoma show positive for p27. (Original magnification, ×400).

).

Although weak in comparison with the nuclei, p27 staining in the cytoplasm was also observed in 67.1% of G1 cases (49 out of 73 cases), 61.5% of G2 (16 out of 26 cases) and 25.0% of G3 (seven out of 28 cases), respectively.

### The correlation of p27 expression with Ki-67 expression

**Figure 3:**
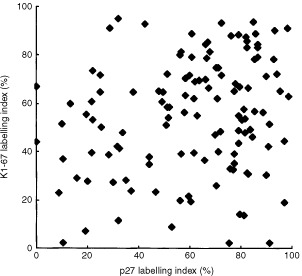
Correlation of p27 expression with Ki-67. LI of p27 was significantly correlated with that of Ki-67 in endometrioid adenocarcinoma of the uterine corpus.

Positive staining of KI-67 was observed in the nuclei of endometrioid adenocarcinomas of the uterine corpus. LI of p27 expression was significantly correlated with that of Ki-67 (Rs=0.226, *P*=0.017) (Figure 3

).

### The correlation between p27 expression and clinicopathological parameters

The high level of p27 expression was significantly correlated with the clinicopathological parameters such as FIGO stage I *vs* IV (*P*=0.03), II vs IV (*P*=0.048), and III *vs* IV (*P*=0.03), lymph node metastasis (*P*<0.0001), lymphovascular space involvement (LVSI) (*P*<0.0001) and myometrial invasion (*P*=0.042). In contrast, it was not correlated with Group (Group 1: coexisting with endometrial hyperplasia, Group 2: coexisting with normal endometrium, Group 3: entirely replaced by carcinoma) (Ohkawara *et al*, 2000), ER and PR, and menopause (Table 1).

## DISCUSSION

Our study showed that p27 staining was observed in the nuclei of the glandular cells in the secretory phase but negligible in the proliferative phase of normal endometrium. This result is the same as in previous reports (Shiozawa *et al*, 1998; Bamberger *et al*, 1999). Shiozawa *et al* (1998) suggested that a markedly increased p27 expression induced by progesterone in the secretory phase might develop cell growth arrest by inhibiting the cyclin E/cdk2 complex and it was a result of a persistent accumulation of p27 due to a prolonged half-life by progesterone-mediated impaired proteolytic activity.

Surprisingly, p27 expression in endometrioid adenocarcinoma of the uterine corpus increased significantly in the higher histological grades in our study. Two reports on endometrial adenocarcinoma demonstrated that decreased p27 expression was correlated with higher histological grade (Ahn *et al*, 1998; Bamberger *et al*, 1999). Recently, however, a trend associated with increased p27 staining with advanced grades of endometrial carcinoma was reported (Nycum *et al*, 2001). Our study showed that p27 expression was correlated with that of Ki-67 as a proliferative marker. Similar results of a positive correlation between p27 expression and Ki-67 expression were reported in the colon (Cheng *et al*, 1999) and lung (Shoji *et al*, 1999). In some highly proliferative human breast cancer cells (Fredersdorf *et al*, 1997) and Burkitt's lymphoma cells (Barnouin *et al*, 1999), a high level p27 expression was seen. These results indicate that p27 expressed in carcinoma may not arrest the cell cycle progression.

A possible mechanism of p27 expression abnormality could be considered as follows: (1) its functional abnormality may be due to gene mutation. But no detectable cancer-specific mutations were found in a total of 147 human tumours (Ponce-Castaneda *et al*, 1995). Although polymorphism as a nucleotide substitution of guanine for thymine (GTC→GGC) at codon 109 was found in endometrial, uterine cervical and ovarian cancers, this polymorphism is also detected in normal cells (Kawamata *et al*, 1995). Deletions of the p27 gene have only been detected in B-immunoblastic non-Hodgkin's lymphomas and adult T-cell leukaemias/lymphomas (Morosetti *et al*, 1995); (2) There may be a quantitative or structural abnormality of the cyclin E/cdk2 complex. There are two possibilities. One is an excessive amount of the complex beyond the inhibitory action of p27. The other is that p27 may act controversially as an assembly factor to stabilise the complex (Shoji *et al*, 1999); (3) Consumption of p27 may be trapped by other factors such as cyclin D1 and D3, which suppressed the formation of the complex (Fredersdorf *et al*, 1997; Tomoda *et al*, 1999); (4) p27 degradation by the ubiquitin-proteasome pathway in which skp2 is implicated (Carrano *et al*, 1999) may be disordered. Clarification of the precise mechanism of these possibilities, however, is left for a future study. Moreover, p27 may be overexpressed by a homeostatic feedback mechanism in overexpressed cases of p27, since high levels of cyclin E and cdk2 expressions are observed in these cases (Kawana *et al*, 1998; Cheng *et al*, 1999).

p27 expression in the cytoplasm has been reported in oesophageal (Gillett *et al*, 1999), colon (Fredersdorf *et al*, 1997) and ovarian carcinomas (Masciullo *et al*, 1999). Our analysis showed that p27 expression in the cytoplasm of endometrioid adenocarcinoma was reduced in the higher grade. With regard to this mechanism, it is suggested that unstable p27 is translocated from the nucleus to the cytoplasm by binding Jab1 as the transreporter (Tomoda *et al*, 1999). Furthermore, a recent study shows that staining in the cytoplasm rather than in the nuclei is correlated with a recurrence and decreased survival (Singh *et al*, 1998). p27, which is localized in the cytoplasm, may not play a significant role in the prognosis of endometrial carcinoma, since p27 expression in endometrioid adenocarcinoma was predominant in the nuclei.

Our study showed that p27 expression in endometrioid adenocarcinomas was associated with prognostic factors, such as FIGO stage, lymph node metastasis, LVSI and myometrial invasion. There are variable views on the correlation between p27 expression and clinicopathological factors in various tumours. For example, low p27 expression was associated with prognostic factors in oesophageal (Singh *et al*, 1998) and ovarian carcinomas (Masciullo *et al*, 1999). It was also reportedly correlated with poor prognosis in colon (Loda *et al*, 1997) and breast carcinomas (Gillett *et al*, 1999) by univariate or multivariate analysis. In contrast, increased p27 expression was associated with poor prognosis in oesophageal (Anayama *et al*, 1998) and colon cancers (Fredersdorf *et al*, 1997). The majority of reports suggest that either high or low p27 expression is an independent prognostic factor. In endometrial carcinoma, it was reported that p27 expression was not associated with prognostic factors (Ahn *et al*, 1998; Bamberger *et al*, 1999; Nycum *et al*, 2001), whereas in our study the high expression was correlated with prognostic factors, since p27 protein expression increased in high grade and was associated with cell proliferation in our study.

p27 expression in endometrioid adenocarcinomas was not associated with hormone-related clinicopathological situations such as menopause, Group (Group 1: coexisting with endometrial hyperplasia, Group 2: coexisting with normal endometrium, Group 3: entirely replaced by carcinoma) (Ohkawara *et al*, 2000), and expression of ER or PR, although it was reportedly mediated by a progesterone-related hormonal environment in normal endometrium (Shiozawa *et al*, 1998). A recent study showed that antiestrogen-induced p21 and p27 via ER blocked entry of G1 phase cells into the S phase by inhibiting cyclin D1 expression and cdk2 kinase activity in breast cancer cell lines (Watts *et al*, 1995). The relation between p27 expression and hormonal regulation in normal and neoplastic endometrial cells is left for further investigation.

In endometrioid adenocarcinoma of the uterine corpus, high p27 expression was linked to higher grade and Ki-67 expression, and may be paradoxical in the physiological action of p27. p27 expression was also correlated with clinical prognostic factors such as FIGO stage, lymph node metastasis, LVSI and myometrial invasion. Therefore, p27 expression might be an indicator of poor prognosis. We need more time to elucidate the relation between our results and our patients' actual prognosis.
